# Open Repair of Thoracoabdominal Aortic Aneurysms in the Endovascular Era: When and for Whom

**DOI:** 10.3390/jcm15114013

**Published:** 2026-05-22

**Authors:** Lucas Ribé, Luis E. Guilarte, Eanas S. Yassa, Anthony L. Estrera, Rana O. Afifi

**Affiliations:** 1 Department of Cardiothoracic and Vascular Surgery, McGovern Medical School at UTHealth Houston, Houston, TX 77030, USA; lucas.ribe@uth.tmc.edu (L.R.); luis.e.guilarte@uth.tmc.edu (L.E.G.); anthony.l.estrera@uth.tmc.edu (A.L.E.); 2 Frederik Meijer Heart and Vascular Institute, Corewell Health, Grand Rapids, MI 49503, USA; eanas.yassa@corewellhealth.org; 3 College of Human Medicine, Michigan State University, Grand Rapids, MI 49503, USA

**Keywords:** thoracoabdominal aortic aneurysms, open repair, endovascular era, descending thoracic aorta, heritable thoracic aortic disorders

## Abstract

Open surgical repair continues to play an essential role in the contemporary management of thoracoabdominal aortic aneurysms, despite the dramatic advances and growing adoption of endovascular aortic techniques, which include the combination of thoracic endovascular aortic repair and complex endovascular aortic repair with fenestrated and branched endovascular repair. The relative advantages and limitations of open vs. endovascular approaches must be carefully balanced in each patient. However, open repair of thoracoabdominal aortic aneurysms continues to provide unique advantages in selected patients. This manuscript presents a narrative review of the current literature, addressing both open surgical repair and endovascular approaches in repairing thoracoabdominal aortic aneurysms.

## 1. Introduction

Thoracoabdominal aortic aneurysm (TAAA) is an uncommon and highly complex disease. It is characterized by aneurysmal dilation of the aorta, which extends from the thoracic to the abdominal segments. The yearly incidence of TAAA is 5.9/100,000, and that of acute aortic syndrome is 7/100,000 [1,2]. Those numbers are most likely an underestimation, since TAAA is often silent and can remain asymptomatic for years before becoming symptomatic or being diagnosed.

The central pathophysiologic mechanism in TAAAs is medial degeneration, characterized by elastin fragmentation, smooth muscle cell apoptosis, and proteoglycan accumulation within the extracellular matrix. These alterations reduce elastic recoil and tensile strength, impairing the aorta’s capacity to accommodate pulsatile flow [2,3,4]. Genetic heritable thoracic aortic disorders and familial thoracic aortic syndromes predispose to early medial weakness, while chronic hypertension can increase mechanical stress. Although atherosclerosis is less central than in infrarenal aneurysms, it may exacerbate ischemia of the vasa vasorum, further compromising medial integrity [2,5].

Aortic aneurysm rupture can occur even when the aneurysm diameter is only moderately increased [6]. Previous studies have identified several predisposing factors for aortic rupture, including an aneurysm diameter greater than 5 cm, active or prior smoking, hypertension, chronic obstructive pulmonary disease (COPD), pain, and chronic aortic dissection [7,8,9,10]. In addition to these risk factors, saccular configurations, rapid aneurysm expansion, and the presence of intramural thrombus have been linked to structural instability and accelerated wall stress [8].

Heritable thoracic aortic disorders (HTAD), including Marfan syndrome (MS), Loeys–Dietz syndrome, Ehlers–Danlos syndrome, and ACTA2-related vasculopathies, account for approximately 15–20% of all TAAAs and confer a substantially elevated risk of aortic dissection. Up to 40% of those patients might require thoracoabdominal aortic repair due to aneurysmal enlargement [3,4,5].

The present work aims to provide a comprehensive synthesis of contemporary outcomes associated with both open and endovascular approaches, while, more importantly, delineating the anatomical characteristics and clinical scenarios in which open surgical repair remains the treatment of choice and the most durable treatment strategy.

## 2. Materials and Methods

A narrative review of the English-language literature was conducted to evaluate contemporary outcomes of TAAA repair using open surgical and endovascular techniques. A comprehensive literature search was performed using PubMed/MEDLINE, Embase, and the Cochrane Library to identify studies reporting outcomes of open and endovascular TAAA repair. The search strategy combined Medical Subject Headings (MeSH) and free-text terms, including “thoracoabdominal aortic aneurysm,” “open repair,” “endovascular repair,” and “branched and fenestrated endografts.” Selected articles were manually reviewed to identify additional pertinent studies. We provide a comprehensive, critical, and clinically relevant synthesis of the available evidence published between January 2000 and January 2026.

Inclusion criteria comprised original studies reporting perioperative and/or long-term outcomes in adult patients undergoing TAAA repair, as well as comparative studies, meta-analyses, observational studies, and systematic reviews published between 2000 and 2025. Case reports, non-English publications, editorials, and studies including fewer than 12 patients were excluded. Studies reporting data on hybrid repair, those including thoracic aortic aneurysms alone, or those reporting exclusively ruptured TAAAs, were also excluded. Studies focused exclusively on descending thoracic aneurysms were excluded because they do not involve the complex visceral and renal segment reconstruction that defines TAAA repair and, therefore, differ substantially in operative complexity, perioperative risk, and long-term outcomes. In addition, series centered on ruptured TAAA were not considered, as the emergent nature of presentation introduces profound selection bias and markedly different mortality and morbidity profiles, which are not representative of elective or semi-elective cases.

Data extracted included study design, patient demographics, aneurysm extent, operative details, and major clinical outcomes, such as mortality, spinal cord ischemia, stroke, renal failure, 30-day mortality and survival rates.

The study selection process was conducted in a structured, sequential manner. Two authors (LR, LG) conducted an initial screening of titles and abstracts to identify studies that met the predefined inclusion criteria. Two senior authors (LR and RA) independently performed the final eligibility assessment of all potentially relevant studies. Discrepancies at any stage were resolved through discussion and consensus between the first and senior authors (LR, RA).

### 2.1. Classification of TAAAs

**Figure 1:**
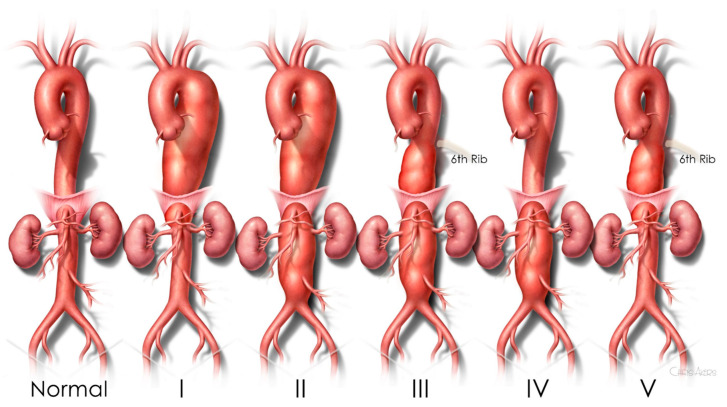
Modified Crawford classification of thoracoabdominal aortic aneurysms. Extent I: Left subclavian artery (LSCA) to above renal arteries; Extent II: LSCA to distal of renal arteries; Extent III: Distal of thoracic level 6 to distal of renal arteries; Extent IV: Distal of thoracic level 12 to distal of renal arteries; and Extent V: distal of thoracic level 6 to proximal of renal arteries.

TAAAs encompass both the descending thoracic aorta (DTA) and the abdominal aorta, extending from just distal to the origin of the left subclavian artery (LSA) to the aortic bifurcation. They are categorized according to the anticipated extent of open surgical repair, using the Crawford and Safi classification system [11,12]. Extent I extends from the LSA (proximal to the sixth rib) to just above the renal arteries. Extent II repairs extend from the LSA to below the renal arteries. Extent III starts distal to the sixth rib, above the diaphragm, to below the renal arteries into the abdominal aorta. Extent IV begins below the level of the diaphragm and extends to below the renal arteries (including the visceral aortic segment). Extent V includes from the sixth intercostal space to just above the renal arteries [12] (Figure 1).

TAAAs are among the most challenging conditions encountered by cardiovascular surgeons. Surgical indications are primarily determined by aneurysm diameter, growth rate, symptomatology, and patient-specific considerations.

Current consensus guidelines from the Society for Vascular Surgery and the American Heart Association/American College of Cardiology Joint Committee on Clinical Practice Guidelines American Heart Association (AHA) generally recommend repair when the maximum diameter reaches approximately 6.0 cm for degenerative TAAAs. However, some high-volume aortic centers advocate for earlier intervention (at around 5.5 cm), particularly in patients with smaller body habitus, in women, or when imaging demonstrates features suggesting structural instability [2,13].

### 2.2. Perioperative Evaluation

When performing the preoperative evaluation and imaging assessment of patients with TAAAs, a careful and comprehensive evaluation of the patient’s overall clinical status is essential. All available imaging studies should be reviewed and compared with prior examinations, including those obtained at the treating institution and at outside hospitals. Given the variable progression of the disease over time, both a baseline study and the most recent imaging study should be carefully assessed and compared, when available.

The preoperative risk evaluation typically requires a systemic work-up that includes cardiac function, pulmonary function, renal function and occlusive disease of the carotid and peripheral arteries workup [14]. In addition, we recommend frailty evaluation, since it has been associated with higher morbidity and mortality in patients undergoing aortic aneurysm repair [15,16,17]. It is essential to have anatomical information to help with surgical planning. Computed tomography angiography (CTA) is the gold standard. Alternative imaging modalities include magnetic resonance imaging (MRI), which should be considered if CTA is contraindicated [14].

### 2.3. Open Surgical and Endovascular Techniques

#### 2.3.1. Open Surgical Technique

**Figure 2:**
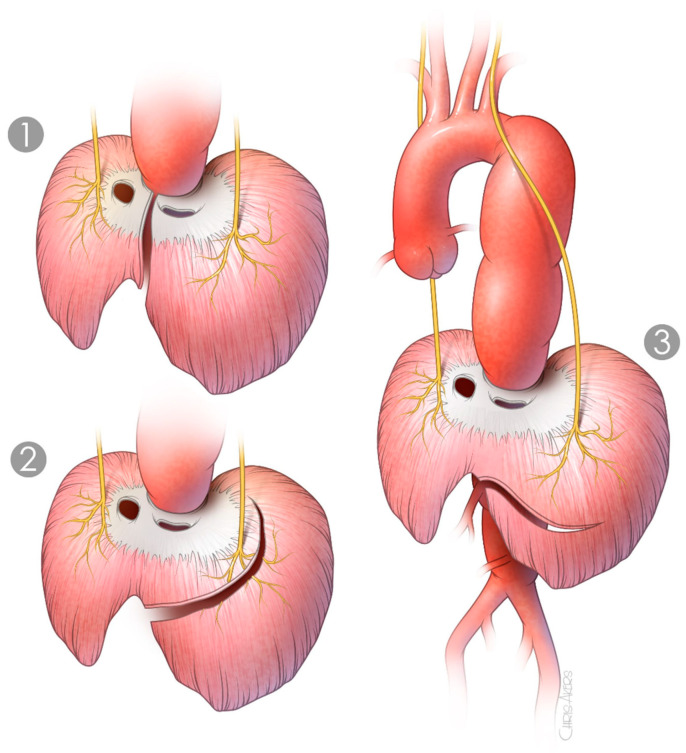
Techniques of diaphragmatic division. (1) Radial diaphragmatic division; (2) lateral diaphragmatic division; and (3) limited lateral diaphragmatic division. A limited lateral division of the diaphragm, performed with preservation of the phrenic nerve, has been associated with improved postoperative recovery of diaphragmatic excursion and pulmonary function.

The technique begins with patient positioning in right lateral decubitus. A left thoracoabdominal incision is used to access the descending thoracic aorta, diaphragm, and retroperitoneal abdominal aorta. Special care is taken to avoid injury to the phrenic nerve to preserve the pulmonary function (Figure 2). During exposure of the proximal DTA, meticulous attention is directed toward preserving the left recurrent laryngeal nerve.

**Figure 3:**
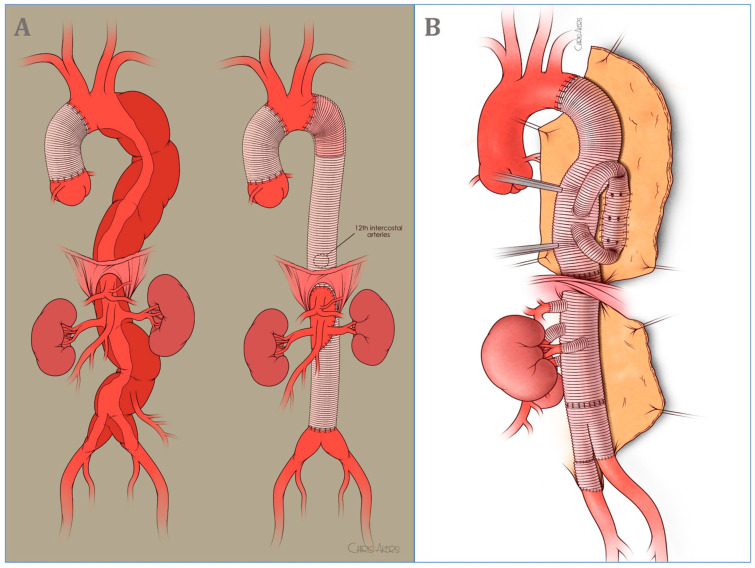
Illustrations showing the two principal surgical strategies for visceral artery revascularization. (**A**) Single-island patch incorporating all target vessels and sewn onto the Dacron graft; and (**B**) separate bypasses to each visceral artery (STAG graft in this image).

Two principal surgical strategies are used for visceral artery revascularization: (1) the creation of a single island patch incorporating all target vessels and sewn onto the Dacron graft; or (2) the construction of separate bypasses to each visceral artery. The choice of configuration depends on several factors, including patient age, the quality of the native aortic tissue, the presence of heritable thoracic aortic disease, and the anatomic distance between the origins of the visceral arteries [18,19] (Figure 3).

Different adjuncts are used to lower spinal cord injury risk, such as the placement of a lumbar drain, distal aortic perfusion with left heart bypass, and neuromonitoring. Open surgical technique has been described in detail in previous manuscripts and is beyond the scope of this review [18,19,20,21].

#### 2.3.2. Endovascular Technique

Endovascular techniques for TAAA repair require advanced fluoroscopic imaging, with preference for hybrid operating room capabilities. An endovascular approach avoids thoracotomy and extracorporeal circulation, with total endovascular repair possible through vascular access via lower and/or upper extremities. These procedures may be performed using various devices and approaches, including fenestrated and branched endovascular aortic repair (FB-EVAR), custom-made fenestrated endovascular aortic repair, physician-modified endografts, and in situ laser fenestration. In most centers, access to some of these devices remains limited. Detailed descriptions of the different techniques have been described previously and are beyond the scope of this review [22,23].

## 3. Results

During the review period (2000–2025), the literature search identified 234 potentially eligible studies. After application of the exclusion criteria, 21 manuscripts on open TAAA repair were selected. For endovascular TAAA repair, 15 manuscripts were included. These studies are summarized in Table 1 and Table 2.

### 3.1. Outcomes of Open Repair

Open repair remains the standard treatment for treating complex TAAAs [26]. Although it may carry a substantial perioperative risk, it offers a definitive solution by replacing the diseased aortic segment and preventing rupture [20]. Despite improvement in perioperative care, mortality and morbidity remain significant, with mortality reported up to 19.8% and morbidity up to 38%. Outcomes are summarized in Table 1 [20,24,25,26,27,28,29,30,31,32,33,34,35,36,37,39,40,41,42,43]. The number of patients included in each study varies widely, ranging from 12,245 in a large meta-analysis by Khan et al. [24] to 28 patients in a single-center experience by Kazen et al. [43]. The largest single-center series were published by Coselli et al. with 3309 patients [25], followed by Estrera et al. with 1896 patients [26].

There is substantial heterogeneity among the available studies, limiting the ability to perform direct, meaningful comparisons. In addition, many reports originate from the same institutions over time and focus on different patient subgroups and research questions. Therefore, rather than attempting direct comparisons, we highlight key findings from these studies to provide context and insight into the evolving evidence.

Operative mortality for open repair of TAAA ranges from 2.2% to 19.8%, depending on the center’s expertise and patient population. Postoperative complications included stroke (1.3 to 12%), spinal-cord ischemia (2 to 13.5%), and renal failure (1.1 to 34.7%) [24]. Although morbidities remain considerable after open repair, outcomes appear better in high-volume, experienced centers [24,27,57].

The largest series on open TAAA repair to date included 3309 TAAA repairs. Coselli et al. reported operative mortality of 7.5%, permanent paraplegia and paraparesis in 2.9% and 2.4%, respectively, and 5.7% permanent renal failure [25].

Once patients survive the postoperative period, long-term survival after elective open repair is relatively favorable. One study by Shimamura et al., which included a cohort of 393 elective open repairs (Crawford extents I-III) between 2003 and 2015, reported survival of 90% at 3 months, 84% at 1 year, 78% at 5 years, and 75% at 10 years. However, those with post-dissection (vs. degenerative) aneurysms experienced significantly higher mortality [34].

Outcomes are better in younger patients, most likely due to lower rates of comorbidities. Coselli et al. report 2.2% early mortality in patients 50 years old and younger [32]. Tanaka et al. reported operative mortality of 6% vs. 17% (*p* < 0.001) in patients younger than 50 years old compared to the older cohort [38].

### 3.2. Outcomes of Endovascular Repair

While complex endovascular repair of intact TAAAs has demonstrated favorable outcomes in highly experienced centers, its application in ruptured TAAAs is limited by the frequent hemodynamic instability of these patients [45,56,58].

Multiple studies have reported that FB-EVAR is both safe and effective for degenerative TAAAs, achieving outcomes competing with those from traditional open repair at high-volume centers [22,23,33,44,45,46,48,49,50,53,55,59] (Table 2). A recent report by Oikonomou and colleagues analyzed outcomes in 71 patients treated with FB-EVAR for TAAAs [51]. Over a mean follow-up period of 25 months, they achieved a technical success rate of 96%, observed a 30-day mortality of 6%, and documented both dialysis dependence and paraplegia in 3% of patients [51].

A 2023 multicenter study, which included 2603 patients who underwent FB-EVAR for TAAAs, reported significantly worse outcomes among those treated non-electively. Early mortality was markedly higher in the nonelective group compared with the elective cohort (17% vs. 5%, *p* < 0.001), as were rates of major adverse events (34% vs. 20%, *p* < 0.001). Long-term outcomes showed a similar pattern. At 3 years, overall survival (50 ± 4% vs. 70 ± 1%) was significantly worse in non-elective patients (*p* < 0.001) [44]. In a post-dissection TAAA (PD-TAAA) group study, 36-month survival was 72%, while target-vessel patency remained high (94%), and no aneurysm ruptures occurred during follow-up [52].

## 4. Discussion

Comparative studies of open vs. endovascular repair for TAAAs may be subject to substantial confounding, complicating the interpretation of outcomes. Selection bias is inherent to most observational series. Patients offered endovascular repair are frequently older, more comorbid, and considered physiologically high risk for open surgery, whereas younger and fitter individuals are preferentially selected for open repair. Conversely, patients with anatomy unsuitable for endovascular treatment (e.g., inadequate landing zones, severe visceral vessel angulation, extensive mural thrombus, or limited access vessels) are systematically directed toward open reconstruction [32].

Anatomic heterogeneity further confounds comparisons. The Crawford extent of aneurysm, prior aortic operations, and branch vessel complexity vary considerably between cohorts, yet are inconsistently reported. Urgency of presentation also differs, as endovascular strategies are often used in frail patients and urgent/symptomatic cases, whereas open repair is more commonly performed electively in stable candidates.

Outcomes are additionally influenced by institutional experience. Both open and complex FB-EVAR demonstrate strong volume–outcome relationships, with high-volume aortic centers reporting lower morbidity and mortality [26]. Finally, device generation introduces temporal bias. Early endograft platforms were associated with higher rates of endoleak and reintervention, whereas contemporary branched and fenestrated systems have improved technical success [45].

Careful stratification by center experience and device platform, transparent reporting of anatomic complexity and urgency, and risk adjustment are essential when interpreting open vs. endovascular TAAA data.

A population-based study by Rocha et al. reported that open repair was associated with higher in-hospital mortality (17.4% vs. 10.8%, *p* = 0.04), longer length of stay, more adverse events, and more frequent discharge to rehabilitation than endovascular repair [35]. Moreover, a short-term meta-analysis of comparative studies found that endovascular repair of TAAA had lower early mortality, lower incidence of renal failure requiring dialysis, and shorter hospital stays [60].

A 2022 meta-analysis, which included 12 observational studies, compared open vs. endovascular TAAA repair and found that endovascular approaches were associated with lower all-cause mortality (HR = 1.91, favoring EVAR), lower risk of spinal cord ischemia, and fewer respiratory and cardiac complications [61]. On the other hand, a systematic review of 71 contemporary studies (24 EVAR, 47 open) showed that rates of permanent paraplegia were similar between open and endovascular repair, with similar in-hospital mortality [62].

One of the strongest arguments in favor of open repair is its superior long-term durability. A 2022 propensity-matched study comparing open vs. endovascular repair of descending thoracic and thoracoabdominal aneurysms reported that, although the in-hospital mortality rates were similar (8.3% vs. 7.6%), open repair conferred significantly better 10-year survival (52% vs. 33%) and a much lower rate of aortic reintervention (4% vs. 21%) in the endovascular group [37]. A systematic review of DTAs similarly found that open repair achieved better long-term, all-cause survival beyond a certain time point, and freedom from reintervention was superior in the open cohort [63].

In response to these challenges, centers of excellence in aortic surgery have refined open surgical techniques and patient selection to improve their outcomes. Strategies include staged repair, rigorous spinal cord protection (cerebrospinal fluid drains, left-heart bypass, and neurophysiological monitoring), organ perfusion strategies, and advocacy for high-volume, specialized centers [47,64,65].

### When Should Open Repair Be Considered First?

Hereditary thoracic aortic disease (HTAD)—Despite the increase in the utilization of endovascular repair for aortic pathologies in patients with HTAD, open TAAA repair remains the gold standard strategy for many patients with these conditions, offering the most reliable and durable treatment option [45,58,59]. Open repair enables the surgeon to excise the diseased aorta, reimplant visceral vessels directly, and replace it with a durable, prosthetic graft, providing long-term structural stability. As described previously, HTADs represent a series of conditions that result in changes in the aortic wall and predispose to aortic dissections or aneurysmal degeneration, such as MS, Loeys–Dietz syndrome, vascular Ehlers–Danlos syndrome, ACTA mutations, and other related genetic aortopathies [2,11,13]. Recent reports from both single-center and multicenter cohorts highlight the strong performance of open procedures, especially when performed in high-volume institutions. Across these studies, investigators consistently report low operative mortality (4%), favorable long-term survival (83% at 5 years), and acceptable complication profiles, even when treating complex TAAAs or performing redo operations [2,36,66,67].

However, there is increased utilization of endovascular repair in HTAD. It is associated with a markedly elevated likelihood of retrograde type A aortic dissection, with reported occurrence rates reaching 25% [67,68]. Dong et al. reported a significantly higher incidence of stent graft-induced new entry (SINE) in patients with MS following type B aortic dissection, occurring in 33.33% of Marfan patients compared with 3.26% of non-Marfan patients (*p* = 0.016) [69].

**Younger patients—**Open TAAA repair remains the preferred treatment for young patients with extensive post-dissection TAAA or aneurysmal disease because of its long-term durability, ability to remove all (or most) of the diseased aortic tissue, and reduced need for reintervention compared with endovascular approaches. Younger patients typically have a longer life expectancy and a lower comorbid disease burden, making durable aortic reconstruction an essential priority and a feasible option with a reasonable recovery potential. Open repair allows replacement of the diseased thoracoabdominal aorta, offers excellent long-term stability, and is less prone to degeneration or failure over the long term [38]. In contrast, complex endovascular repair with FB-EVAR relies on stent graft fixation in native aortic tissue. That tissue is inherently unstable as a long-term landing zone. In younger patients, progressive remodeling, dilation of landing zones, and long-term biomechanical stress may increase the risk of endoleaks, branch instability, and reinterventions, which can accumulate over a patient’s lifetime. Some studies have reported that younger patients (<65 years old) experience higher rates of late reintervention after endovascular repair [38,61].

**Complex anatomy not suitable for endovascular grafts—**Certain anatomic factors may render endovascular repair unsuitable, making open surgical repair the preferred treatment approach. One key limitation of endovascular techniques is the inadequacy of proximal or distal landing zones. Patients with severe aortic tortuosity, aneurysmal involvement of the aortic arch, or a very short or sharply angled proximal landing zone are at increased risk of endoleak, graft migration, and procedural failure [54]. Another unfavorable anatomic consideration is small access vessels, typically less than 7 mm in diameter, though the use of surgical conduits remains an option. The use of these conduits increases the overall procedural complexity. Another involves the visceral artery origins that are too close together, severely angulated, stenotic, dissected, or aneurysmal, preventing accurate fenestration or branch alignment. Such situations increase the risk of target-vessel occlusion, branch instability, or technical failure [54]. Finally, complex post-dissection TAAAs, where there is an especially compressed true lumen, multiple visceral vessels arising from different lumens, or large reentry tears at visceral levels, also limit the feasibility of FB-EVAR. In these settings, open repair in patients with low operative risk continues to offer decisive advantages [70]. Several adjunctive techniques and evolving technologies (such as aortic septotomy and the use of iliac conduits) may help overcome many of these anatomical constraints; however, more data on outcomes and the long-term durability of repairs using those adjuncts are needed.

**Failed prior endovascular repair—**Failure of a prior endovascular repair, including persistent endoleaks, graft migration, structural failure, SINE, or aneurysm expansion, is a well-recognized indication for conversion to open thoracoabdominal aortic repair, with off-label use as the strongest predictor of repair failure [71,72,73].

**Infection—**Graft infections are generally a contraindication to leaving prosthetic material in place, favoring open explant, tissue debridement, and revascularization. The main limitation of endovascular repair is that it excludes the aneurysm but does not remove all infected aortic and periaortic tissues, leaving a potential nidus of infection or a periaortic abscess [73]. On the other hand, open thoracoabdominal aortic repair allows for complete excision of the infected aorta and surrounding infected or necrotic tissues, thorough extensive debridement, and in situ or extra-anatomic reconstruction using antibiotic-impregnated grafts or cryopreserved allografts. Open repair also provides an excellent option for covering the new aortic graft with biologic conduits, using soft-tissue coverage with omentum, latissimus dorsi, or serratus anterior muscle flaps to minimize reinfection risk [74,75,76].

**Aorto-bronchial and aorto-esophageal fistulas—**The presence of a fistula communicating with a hollow viscus implies contamination and a high likelihood of infection and should be managed as an infected process. Both aortobronchial fistulas (ABF) and aortoesophageal fistulas (AEF) may present massive hemorrhage and eventually develop sepsis. Although thoracic endovascular aortic repair may temporarily stabilize bleeding, it cannot adequately address the essential problems, including the fistulous tract, contaminated or infected tissue and, often, necrotic tissues surrounding mediastinal structures. With the contaminated fistula tract in place, the stent graft becomes exposed to the airway or esophageal lumen, creating a high risk for graft infection, persistent mediastinitis, ongoing sepsis, or fistula recurrence [77]. Open repair allows complete excision of the diseased aorta, debridement of infected and necrotic tissues, resection or repair of the esophageal or bronchial lesion, and reconstruction of the aorta, airway, and gastrointestinal tract under direct vision in the same surgical field. This radical approach offers a realistic chance for long-term eradication of infection and durable closure of the fistulae. A multidisciplinary approach is recommended for all cases of ABF and AEF to ensure rapid hemorrhage control, comprehensive aortic reconstruction, complete excision of an infected aortic graft, repair of esophageal or airway defects, and appropriate administration of antibiotic therapy [78].

**Ruptured TAAAs—**According to the 2022 AHA/American College of Cardiology Joint Committee guidelines, open repair is recommended for patients with ruptured TAAAs requiring intervention. This is recommended, with a class of recommendation of 1 (strong agreement) and a moderate level of evidence [2]. However, the presence of rupture markedly increases operative risk for TAAAs. With reported perioperative mortality of up to 34.2% [57,79], the same guidelines recommend considering endovascular repair in hemodynamically stable patients with suitable anatomy and at centers with the needed expertise and devices [2].

**Figure 4:**
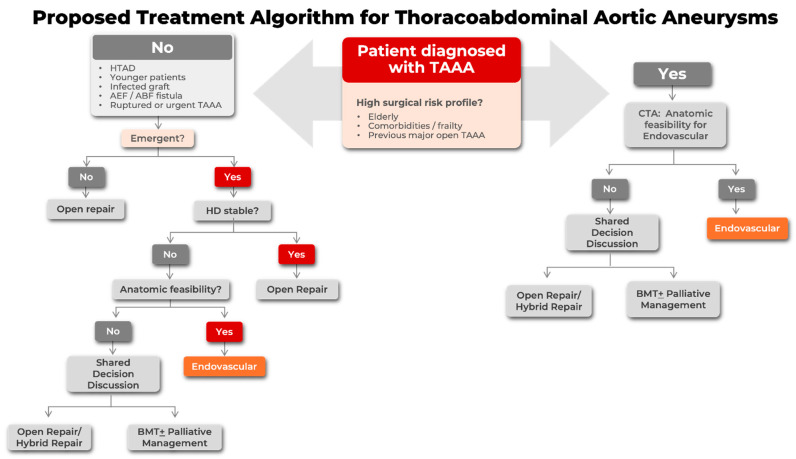
Illustration of a proposed algorithm guiding decision-making for open vs. endovascular management of thoracoabdominal aortic aneurysms.

Based on our institutional experience and a review of the existing literature, we propose an algorithm to guide decision-making between open and endovascular management of TAAAs (Figure 4).

## 5. Limitations

Several limitations were identified in our study. First, the inclusion of both open surgical and endovascular repair series resulted in substantial heterogeneity regarding patient selection, aneurysm extent, operative technique, perioperative management, and outcome reporting. Additional variability in study design, complication definitions, follow-up duration, and the inability to uniformly control for the actual anatomical complexity within each series further limit the comparability of reported outcomes. Referral bias is also likely, as patients treated at high-volume or tertiary institutions are often selectively referred due to greater disease complexity, younger age, or perceived suitability for advanced interventions. The technological advances in the endovascular field make it difficult to compare older studies with contemporary practice. Excluding case reports, non-English publications, editorials, and small series (<12 patients) may reduce publication bias and omit relevant early or niche experiences. Last, the predominance of retrospective studies limits the overall level of evidence. In addition, the proposed decision-making algorithm is based on narrative synthesis and institutional experience rather than formal validation.

## 6. Conclusions

The management of TAAA remains one of the most challenging problems faced by cardiothoracic and vascular surgeons. Open surgical repair remains the preferred approach in selected patients, including those with HTAD, those younger than 50 years of age, those with highly complex anatomy not amenable to endovascular repair, and, in certain cases, those with failed prior endovascular intervention. In addition, open repair remains the treatment of choice for many patients presenting with aortic infection, ABF, or AEF. A comprehensive approach to complex aortic disease mandates a team capable of performing the full breadth of aortic surgery and maintaining proficiency in open techniques. As care is increasingly centralized for complex open repair and decentralized for complex endovascular repair with newer, “off-the-shelf” endovascular devices, it is the responsibility of our medical teams to ensure care is customized for the patient and that equal competency is available to patients who need open or endovascular thoracoabdominal surgery.

## Figures and Tables

**Table 1:** Current open TAAA series, morbidity and survival.

Study	Year	Number Patients	HTAD (%)	Renal Failure (%)	Spinal Cord Ischemia (%)	Stroke (%)	Mortality 30-Day (%)	Survival 1 Year (%)
Khan et al. [24]	2020	12,245	-	1616 (13.2)	1065 (8.7)	600 (4.9)	1285 (10.5)	
Coselli et al. [25]	2016	3309	523 (15.8)	406 (12.2)	317 (9.5)	98 (2.9)	159 (4.8)	83.5%
Estrera et al. [26]	2015	1896	106 (5.6)	461 (24.3)	183 (9.6)	95 (5.0)	302 (15.9)	
Kiser et al. [27]	2022	1474	164 (17%)	261 (27%)	76 (8%)	43 (4%)	134 (14%)	
Coselli et al. [28]	2020	1114	242 (21.7)	193 (17.3)	151 (13.5)	52 (4.6)	116 (10.4)	75.8%
LeMaire et al. [29]	2012	823	111 (13.5%)	45 (5.5%)	42 (5.1%)	27 (3.3%)	69 (8.4)	
Dayama et al. [30]	2015	682	-	80 (11.7)	-	17 (2.5)	68 (9.9)	
Murana et al. [31]	2015	542	37 (6.8)	23 (4.2)	32 (5.9)	23 (4.2)	46 (8.4)	
Coselli et al. [32]	2017	445	237 (53.2)	7 (1.5)	9 (2)	6 (1.3)	10 (2.2)	
Locham et al. [33]	2018	398	-	138 (34.7)	31 (7.8)	26 (6.5)	59 (14.8)	
Shimamura et al. [34]	2019	393	30 (7.6)	18 (4.5)	42 (10.6)	32 (8.1)	24 (6.1)	
Rocha et al. [35]	2021	361	-	62 (17.2)	13 (3.6)	18 (4.9)	64 (17.7)	
LeMaire et al. [36]	2006	300	300 (100)	8 (6)		2 (2)	6 (5)	
Tong et al. [37]	2022	278	277 (99.6)	24 (8.6)	22 (7.9)	15 (5.4)	23 (8.2)	
Tanaka et al. [38]	2019	276	147 (53)	34 (12)	9 (3)	10 (4)	16 (6)	88%
Frankel et al. [39]	2020	155	155 (100)	5 (3.2)	4 (2.6)	2 (1.3)	4 (2.5)	
Frankort et al. [40]	2025	91	24 (26.3)	1 (1.1)	6 (6.6)	11 (12)	18 (19.8)	
Aftab et al. [41]	2015	88	-	20 (22.7)	9 (10.2)	5 (5.7)	12 (13.6)	
De Rango et al. [20]	2011	55	20 (40)	19 (38)	1 (2)	1 (2)	6 (12)	
Mkalaluh et al. [42]	2018	38	18 (47.3)	9 (23.6)	3 (7.9)	3 (7.9)	3 (7.9)	
Kazen et al. [43]	2016	28	6 (21.4)	8 (28.5)	4 (14.2)	1 (3.5)	0	

HTAD: heritable thoracic aortic disease. TAAA: thoracoabdominal aortic aneurysm.

**Table 2:** Current endovascular TAAA series, morbidity and survival.

Study	Year	Number Patients	HTAD (%)	Renal Failure (%)	Spinal Cord Ischemia (%)	Stroke (%)	Mortality 30-Day (%)	Survival 1 Year (%)
Dias-Neto et al. [44]	2023	2603	62 (2.3)	317 (12.1)	278 (10.6)	84 (3.2)	169 (6.4)	-
Oderich et al. [45]	2024	1109	-	111 (10.0)	34 (3.1)	30 (2.7)	30 (2.7)	-
Locham et al. [33]	2018	481	-	65 (13.5)	14 (2.9)	11 (2.2)	26 (5.4)	-
Greenberg et al. [46]	2010	406	-	5 (1.2)	17 (4.3)	-	23 (5.7)	84%
Muston et al. [47]	2023	402	7 (1.7)	8 (2)	39 (9.8)	1 (0.2)	14 (3.6)	-
Eagleton et al. [48]	2016	354	-	18 (5.1)	31 (8.7)	8 (2.2)	17 (4.8)	78%
Tenorio et al. [23]	2019	240	5 (2)	19 (8)	14 (6)	2 (1)	6 (3)	89%
Schanzer et al. [49]	2017	100	-	5 (5)	1 (1)	0	3 (3)	87%
Mahmood et al. [50]	2025	95	16 (16.8)	6 (6.3)	18 (18.9)	5 (5.2)	9 (9.4)	-
Oikonomou et al. [51]	2019	71	3 (4.2)	2 (2.8)	3 (4.2)	0	3 (5.6)	94%
Marques de Marino et al. [52]	2020	55	-	2 (3.6)	6 (10.9)	0	9 (16.4)	87%
Mascoli et al. [53]	2018	39	-	3 (7.7)	4 (10.2)	0	10 (2.5)	72%
Chuter et al. [54]	2011	28	-	0 (0)	1 (3.5)	0	0 (0)	100%
Spear et al. [55]	2018	24	1 (4.1)	2 (8.3)	3 (12.5)	0	1 (4.1)	90%
Ribé et al. [56]	2025	12	12 (100%)	0 (0)	1 (8.3)	0	0 (0)	93%

HTAD: heritable thoracic aortic disease. TAAA: thoracoabdominal aortic aneurysm.

## Data Availability

This study is a narrative review of the published literature. All data discussed in this manuscript are derived from articles available in the public domain. No individual patient-level data were accessed or generated.

## Glossary of Terms

ABF	Aortobronchial fistulas
AEF	Aortoesophageal fistulas
AHA	American Heart Association
COPD	Chronic obstructive pulmonary disease
DTA	Descending thoracic aorta
FB-EVAR	Fenestrated and branched endovascular aortic repair
HTAD	Heritable thoracic aortic disorders
LSA	Left subclavian artery
MS	Marfan syndrome
SINE	Stent graft-induced new entry
STAG	Side-branched Thoracoabdominal Aortic Graft
TAAA	Thoracoabdominal aortic aneurysm

## References

[1] Olsson C., Thelin S., Stahle E., Ekbom A., Granath F. (2006). Thoracic aortic aneurysm and dissection: Increasing prevalence and improved outcomes reported in a nationwide population-based study of more than 14,000 cases from 1987 to 2002. Circulation.

[2] Isselbacher E.M., Preventza O., Black J.H., Augoustides J.G., Beck A.W., Bolen M.A., Braverman A.C., Bray B.E., Brown-Zimmerman M.M., Chen E.P. (2022). 2022 ACC/AHA Guideline for the Diagnosis and Management of Aortic Disease: A Report of the American Heart Association/American College of Cardiology Joint Committee on Clinical Practice Guidelines. Circulation.

[3] Pinard A., Jones G.T., Milewicz D.M. (2019). Genetics of Thoracic and Abdominal Aortic Diseases. Circ. Res..

[4] Zafar M.A., Chen J.F., Wu J., Li Y., Papanikolaou D., Abdelbaky M., Vinholo T.F., Rizzo J.A., Ziganshin B.A., Mukherjee S.K. (2021). Yale Aortic Institute Natural History Investigators. Natural history of descending thoracic and thoracoabdominal aortic aneurysms. J. Thorac. Cardiovasc. Surg..

[5] Kim J.B., Kim K., Lindsay M.E., MacGillivray T., Isselbacher E.M., Cambria R.P., Sundt T.M. (2015). Risk of rupture or dissection in descending thoracic aortic aneurysm. Circulation.

[6] Oda T., Minatoya K., Sasaki H., Tanaka H., Seike Y., Itonaga T., Inoue Y., Higashi M., Nishimura K., Kobayashi J. (2017). Surgical Indication for Chronic Aortic Dissection in Descending Thoracic and Thoracoabdominal Aorta. Circ. Cardiovasc. Interv..

[7] Estrera A.L., Sandhu H.K., Miller C.C., Charlton-Ouw K.M., Nguyen T.C., Afifi R.O., Azizzadeh A., Safi H.J. (2014). Repair of extensive aortic aneurysms: A single-center experience using the elephant trunk technique over 20 years. Ann. Surg..

[8] Juvonen T., Ergin M.A., Galla J.D., Lansman S.L., Nguyen K.H., McCullough J.N., Levy D., A de Asla R., A Bodian C., Griepp R.B. (1997). Prospective study of the natural history of thoracic aortic aneurysms. Ann. Thorac. Surg..

[9] Griepp R.B., Ergin M.A., Galla J.D., Lansman S.L., McCullough J.N., Nguyen K.H., Klein J.J., Spielvogel D. (1999). Natural history of descending thoracic and thoracoabdominal aneurysms. Ann. Thorac. Surg..

[10] Fann J.I. (2002). Descending thoracic and thoracoabdominal aortic aneurysms. Coron. Artery Dis..

[11] Crawford E.S., Crawford J.L., Safi H.J., Coselli J.S., Hess K.R., Brooks B., Norton H.J., Glaeser D.H. (1986). Thoracoabdominal aortic aneurysms: Preoperative and intraoperative factors determining immediate and long-term results of operations in 605 patients. J. Vasc. Surg..

[12] Safi H.J., Winnerkvist A., Miller C.C., Iliopoulos D.C., Reardon M.J., Espada R., Baldwin J.C. (1998). Effect of extended cross-clamp time during thoracoabdominal aortic aneurysm repair. Ann. Thorac. Surg..

[13] Chaikof E.L., Dalman R.L., Eskandari M.K., Jackson B.M., Lee W.A., Mansour M.A., Mastracci T.M., Mell M., Murad M.H., Nguyen L.L. (2018). The Society for Vascular Surgery practice guidelines on the care of patients with an abdominal aortic aneurysm. J. Vasc. Surg..

[14] Ogino H., Iida O., Akutsu K., Chiba Y., Hayashi H., Ishibashi-Ueda H., Kaji S., Kato M., Komori K., Matsuda H. (2023). JCS/JSCVS/JATS/JSVS 2020 Guideline on Diagnosis and Treatment of Aortic Aneurysm and Aortic Dissection. Circ. J..

[15] Zhang J., Qiu Y., Zhang H., Fan Y. (2024). Impact of frailty on adverse outcomes in patients with abdominal aortic aneurysm undergoing surgery: A systematic review and meta-analysis. J. Nutr. Health Aging.

[16] Xu F., Mo J., Chen X. (2025). Role of Frailty in Predicting Outcomes After Surgical Repair of Abdominal Aortic Aneurysm: A Systematic Review and Meta-Analysis. Ann. Vasc. Surg..

[17] Percy E.D., Vinholo T.F., Newell P., Singh S., Hirji S., Awtry J., Semco R., Chowdhury M., Reed A.K., Asokan S. (2024). The Impact of Frailty on Outcomes of Proximal Aortic Aneurysm Surgery: A Nationwide Analysis. J. Cardiovasc. Dev. Dis..

[18] Afifi R.O., Tanaka A., Yazji I., Safi H.J., Estrera A.L. (2017). Thoracoabdominal aortic aneurysm repair in Marfan syndrome: How we do it. Ann. Cardiothorac. Surg..

[19] Ouzounian M., Tadros R.O., Svensson L.G., Lyden S.P., Oderich G.S., Coselli J.S. (2022). Thoracoabdominal Aortic Disease and Repair: JACC Focus Seminar, Part 3. J. Am. Coll. Cardiol..

[20] De Rango P., Estrera A.L., Miller C.C., Lee T.Y., Keyhani K., Abdullah S., Safi H. (2011). Operative outcomes using a side-branched thoracoabdominal aortic graft (STAG) for thoracoabdominal aortic repair. Eur. J. Vasc. Endovasc. Surg..

[21] Tanaka A., Smith H.N., Safi H.J., Estrera A.L. (2023). Open Treatments for Thoracoabdominal Aortic Aneurysm Repair. Methodist DeBakey Cardiovasc. J..

[22] Oderich G.S., Ribeiro M.S., Sandri G.A., Tenorio E.R., Hofer J.M., Mendes B.C., Chini J., Cha S. (2019). Evolution from physician-modified to company-manufactured fenestrated-branched endografts to treat pararenal and thoracoabdominal aortic aneurysms. J. Vasc. Surg..

[23] Tenorio E.R., Oderich G.S., Farber M.A., Schneider D.B., Timaran C.H., Schanzer A., Beck A.W., Motta F., Sweet M.P., U.S. Fenestrated and Branched Aortic Research Consortium Investigators (2020). Outcomes of endovascular repair of chronic postdissection compared with degenerative thoracoabdominal aortic aneurysms using fenestrated-branched stent grafts. J. Vasc. Surg..

[24] Khan F.M., Naik A., Hameed I., Robinson N.B., Spadaccio C., Rahouma M., Yongle R., Demetres M., Chen H., Chang M. (2020). Open Repair of Descending Thoracic and Thoracoabdominal Aortic Aneurysms: A Meta-Analysis. Ann. Thorac. Surg..

[25] Coselli J.S., LeMaire S.A., Preventza O., de la Cruz K.I., Cooley D.A., Price M.D., Stolz A.P., Green S.Y., Arredondo C.N., Rosengart T.K. (2016). Outcomes of 3309 thoracoabdominal aortic aneurysm repairs. J. Thorac. Cardiovasc. Surg..

[26] Estrera A.L., Sandhu H.K., Charlton-Ouw K.M., Afifi R.O., Azizzadeh A., Miller C.C., Safi H.J. (2015). A Quarter Century of Organ Protection in Open Thoracoabdominal Repair. Ann. Surg..

[27] Kiser K.A., Tanaka A., Sandhu H.K., Miller C.C., Leonard S.D., Safi H.J., Estrera A.L. (2022). Extensive cell salvage and postoperative outcomes following thoracoabdominal and descending aortic repair. J. Thorac. Cardiovasc. Surg..

[28] Coselli J.S., Green S.Y., Price M.D., Zhang Q., Preventza O., de la Cruz K.I., Whitlock R., Amarasekara H.S., Woodside S.J., Perez-Orozco A. (2020). Spinal cord deficit after 1114 extent II open thoracoabdominal aortic aneurysm repairs. J. Thorac. Cardiovasc. Surg..

[29] LeMaire S.A., Price M.D., Green S.Y., Zarda S., Coselli J.S. (2012). Results of open thoracoabdominal aortic aneurysm repair. Ann. Cardiothorac. Surg..

[30] Dayama A., Sugano D., Reeves J.G., Rivera A., Tsilimparis N. (2016). Early outcomes and perioperative risk assessment in elective open thoracoabdominal aortic aneurysm repair: An analysis of national data over a five-year period. Vascular.

[31] Murana G., Castrovinci S., Kloppenburg G., Yousif A., Kelder H., Schepens M., de Maat G., Sonker U., Morshuis W., Heijmen R. (2016). Open thoracoabdominal aortic aneurysm repair in the modern era: Results from a 20-year single-centre experience. Eur. J. Cardiothorac. Surg..

[32] Coselli J.S., Amarasekara H.S., Green S.Y., Price M.D., Preventza O., de la Cruz K.I., Zhang Q., LeMaire S.A. (2017). Open Repair of Thoracoabdominal Aortic Aneurysm in Patients 50 Years Old and Younger. Ann. Thorac. Surg..

[33] Locham S., Dakour-Aridi H., Nejim B., Dhaliwal J., Alshwaily W., Malas M. (2018). Outcomes and cost of open versus endovascular repair of intact thoracoabdominal aortic aneurysm. J. Vasc. Surg..

[34] Shimamura J., Oshima S., Ozaki K., Sakurai S., Hirai Y., Hirokami T., Fujikawa T., Ozaki A., Yamamoto S. (2019). Open Thoracoabdominal Aortic Aneurysm Repair: Contemporary Outcomes for 393 Elective Cases. Ann. Thorac. Surg..

[35] Rocha R.V., Lindsay T.F., Austin P.C., Al-Omran M., Forbes T.L., Lee D.S., Ouzounian M. (2021). Outcomes after endovascular versus open thoracoabdominal aortic aneurysm repair: A population-based study. J. Thorac. Cardiovasc. Surg..

[36] LeMaire S.A., Carter S.A., Volguina I.V., Laux A.T., Milewicz D.M., Borsato G.W., Cheung C.K., Bozinovski J., Markesino J.M., Vaughn W.K. (2006). Spectrum of aortic operations in 300 patients with confirmed or suspected Marfan syndrome. Ann. Thorac. Surg..

[37] Tong M.Z., Eagleton M.J., Roselli E.E., Blackstone E.H., Xiang F., Ibrahim M., Johnston D.R., Soltesz E.G., Bakaeen F.G., Lyden S.P. (2022). Outcomes of Open Versus Endovascular Repair of Descending Thoracic and Thoracoabdominal Aortic Aneurysms. Ann. Thorac. Surg..

[38] Tanaka A., Leonard S.D., Sandhu H.K., Afifi R.O., Miller C.C., Charlton-Ouw K.M., Ray A., Hassan M., Safi H.J., Estrera A.L. (2019). Open Descending and Thoracoabdominal Aortic Repairs in Patients Younger Than 50 Years Old. Ann. Thorac. Surg..

[39] Frankel W.C., Song H.K., Milewski R.K., Shalhub S., Pugh N.L., Eagle K.A., Roman M.J., Pyeritz R.E., Maslen C.L., Ravekes W.J. (2020). GenTAC Investigators. Open Thoracoabdominal Aortic Repair in Patients with Heritable Aortic Disease in the GenTAC Registry. Ann. Thorac. Surg..

[40] Frankort J., Keszei A., Doukas P., Uhl C., Jacobs M.J., Mees B.M.E., Gombert A., Elfeky M. (2026). Outcome following open TAAA repair after TEVAR compared to conventional open type II TAAA repair. Vasa.

[41] Aftab M., Songdechakraiwut T., Green S.Y., Zarda S., Price M.D., Nalty C.C., Preventza O., de la Cruz K.I., LeMaire S.A., Coselli J.S. (2015). Contemporary outcomes of open thoracoabdominal aortic aneurysm repair in octogenarians. J. Thorac. Cardiovasc. Surg..

[42] Mkalaluh S., Szczechowicz M., Dib B., Weymann A., Szabo G., Karck M. (2018). Open surgical thoracoabdominal aortic aneurysm repair: The Heidelberg experience. J. Thorac. Cardiovasc. Surg..

[43] Kazen U.P., Blohmé L., Olsson C., Hultgren R. (2016). Open Repair of Aneurysms of the Thoracoabdominal Aorta. Thorac. Cardiovasc. Surg..

[44] Dias-Neto M., Vacirca A., Huang Y., Baghbani-Oskouei A., Jakimowicz T., Mendes B.C., Kolbel T., Sobocinski J., Bertoglio L., Mees B. (2023). Outcomes of Elective and Non-elective Fenestrated-branched Endovascular Aortic Repair for Treatment of Thoracoabdominal Aortic Aneurysms. Ann. Surg..

[45] Oderich G.S., Huang Y., Harmsen W.S., Tenorio E.R., Schanzer A., Timaran C.H., Schneider D.B., Mendes B.C., Eagleton M.J., Farber M.A. (2024). Early and Late Aortic-Related Mortality and Rupture After Fenestrated-Branched Endovascular Aortic Repair of Thoracoabdominal Aortic Aneurysms: A Prospective Multicenter Cohort Study. Circulation.

[46] Greenberg R., Eagleton M., Mastracci T. (2010). Branched endografts for thoracoabdominal aneurysms. J. Thorac. Cardiovasc. Surg..

[47] Muston B.T., Bilbrough J., Bushati Y., Wilson-Smith A.R., Misfeld M., Yan T. (2023). Open, closed or a bit of both: A systematic review and meta-analysis of staged thoraco-abdominal aortic aneurysm repair. Ann. Cardiothorac. Surg..

[48] Eagleton M.J., Follansbee M., Wolski K., Mastracci T., Kuramochi Y. (2016). Fenestrated and branched endovascular aneurysm repair outcomes for type II and III thoracoabdominal aortic aneurysms. J. Vasc. Surg..

[49] Schanzer A., Simons J.P., Flahive J., Durgin J., Aiello F.A., Doucet D., Steppacher R., Messina L.M. (2017). Outcomes of fenestrated and branched endovascular repair of complex abdominal and thoracoabdominal aortic aneurysms. J. Vasc. Surg..

[50] Mahmood D.N., Rocha R., Ouzounian M., Teng Tan K., Forbes S.M., Chung J.C.-Y., Lindsay T.F. (2025). Thoracoabdominal Aortic Aneurysm Repair Using Fenestrated and Branched Endovascular Grafts for High-Risk Patients: Evolving yet Safe. J. Endovasc. Ther..

[51] Oikonomou K., Kasprzak P., Katsargyris A., Marques De Marino P., Pfister K., Verhoeven E.L.G. (2019). Mid-Term Results of Fenestrated/Branched Stent Grafting to Treat Post-dissection Thoraco-abdominal Aneurysms. Eur. J. Vasc. Endovasc. Surg..

[52] De Marques Marino P., Ibraheem A., Gafur N., Verhoeven E.L., Katsargyris A. (2020). Outcomes of fenestrated and branched endovascular aortic repair for chronic post-dissection thoracoabdominal aortic aneurysms. J. Cardiovasc. Surg..

[53] Mascoli C., Vezzosi M., Koutsoumpelis A., Iafrancesco M., Ranasinghe A., Clift P., Mascaro J., Claridge M., Adam D.J. (2018). Endovascular Repair of Acute Thoraco-abdominal Aortic Aneurysms. Eur. J. Vasc. Endovasc. Surg..

[54] Chuter T.A., Hiramoto J.S., Park K.H., Reilly L.M. (2011). The transition from custom-made to standardized multibranched thoracoabdominal aortic stent grafts. J. Vasc. Surg..

[55] Spear R., Hertault A., Van Calster K., Settembre N., Delloye M., Azzaoui R., Sobocinski J., Fabre D., Tyrrell M., Haulon S. (2018). Complex endovascular repair of postdissection arch and thoracoabdominal aneurysms. J. Vasc. Surg..

[56] Ribé L., Ruiter Kanamori L., Schmid B.P., Macedo T.A., Mendes B.C., Maximus S., Huang Y., Nasser F., Oderich G.S. (2025). Outcomes of fenestrated-branched endovascular aortic repair of thoracoabdominal aortic aneurysms in patients with heritable thoracic aortic diseases. JTCVS Tech..

[57] Moulakakis K.G., Antonopoulos C.N., Karaolanis G., Kakisis J., Klonaris C., Lazaris A., Sfyroeras G., Theocharopoulos G., Vasdekis S.N., Geroulakos G. (2019). Outcome of open repair of ruptured thoracoabdominal aortic aneurysms: A systematic review and meta-analysis. Hell. J. Vasc. Endovasc. Surg..

[58] Ribe L., Luis J., Vicente J., Garcia-Pajares R., Vila M., Manuel L. (2011). Endovascular Repair of Thoracic Aortic Emergencies. Diagnosis and Treatment of Abdominal and Thoracic Aortic Aneurysms Including the Ascending Aorta and the Aortic Arch.

[59] Oderich G.S., Ribeiro M., Hofer J., Wigham J., Cha S., Chini J., Macedo T.A., Gloviczki P. (2017). Prospective, nonrandomized study to evaluate endovascular repair of pararenal and thoracoabdominal aortic aneurysms using fenestrated-branched endografts based on supraceliac sealing zones. J. Vasc. Surg..

[60] Rocha R.V., Friedrich J.O., Elbatarny M., Yanagawa B., Al-Omran M., Forbes T.L., Lindsay T.F., Ouzounian M. (2018). A systematic review and meta-analysis of early outcomes after endovascular versus open repair of thoracoabdominal aortic aneurysms. J. Vasc. Surg..

[61] Ellahi A., Shaikh F.N., Kashif H., Khan H., Ali E., Nasim B., Adil M., Huda Z., Liaquat A., Arshad M.S. (2022). Effectiveness of endovascular repair versus open surgery for the treatment of thoracoabdominal aneurysm: A systematic review and meta-analysis. Ann. Med. Surg..

[62] Rocha R.V., Lindsay T.F., Friedrich J.O., Shan S., Sinha S., Yanagawa B., Al-Omran M., Forbes T.L., Ouzounian M. (2020). Systematic review of contemporary outcomes of endovascular and open thoracoabdominal aortic aneurysm repair. J. Vasc. Surg..

[63] Liu J., Gou D., Xu K., Lu Z., Li P., Lei Y., Wang Y., Yang Y., Liu S., Zhu G. (2025). Comparison of short-and long-term outcomes between endovascular and open repair for descending thoracic aortic aneurysm: A systematic review and meta-analysis. Int. J. Surg..

[64] Li S., Gao F., Hu H.O., Shi J., Zhang J. (2020). Risk Factors for Mortality in Patients with Aortoesophageal Fistula Related to Aortic Lesions. Gastroenterol. Res. Pract..

[65] El Beyrouti H., Treede H., Halloum N. (2025). Mechanism and Management of Aorto-Esophageal Fistulation after Thoracic Endovascular Aortic Repair. Ann. Vasc. Surg..

[66] Trans-Atlantic Aortic Research Consortium Investigators (2023). Endovascular repair of intercostal and visceral aortic patch aneurysms following open thoracoabdominal aortic aneurysm repair. J. Thorac. Cardiovasc. Surg..

[67] Shalhub S., Eagle K.A., Asch F.M., LeMaire S.A., Milewicz D.M., Gen T.A.C. (2018). Investigators for the Genetically Triggered Thoracic Aortic Aneurysms and Cardiovascular Conditions (GenTAC) Consortium. Endovascular thoracic aortic repair in confirmed or suspected genetically triggered thoracic aortic dissection. J. Vasc. Surg..

[68] Dong Z.H., Fu W.G., Wang Y.Q., Guo D.Q., Xu X., Ji Y., Chen B., Jiang J.H., Yang J., Shi Z.Y. (2009). Retrograde type A aortic dissection after endovascular stent graft placement for treatment of type B dissection. Circulation.

[69] Dong Z., Fu W., Wang Y., Wang C., Yan Z., Guo D., Xu X., Chen B. (2010). Stent graft-induced new entry after endovascular repair for Stanford type B aortic dissection. J. Vasc. Surg..

[70] Sweet M.P., Fillinger M.F., Morrison T.M., Abel D. (2011). The influence of gender and aortic aneurysm size on eligibility for endovascular abdominal aortic aneurysm repair. J. Vasc. Surg..

[71] Preventza O., Cervera R., Cooley D.A., Bakaeen F.G., Mohamed A.S., Cheong B.Y., Cornwell L., Simpson K.H., Coselli J.S. (2014). Acute type I aortic dissection: Traditional versus hybrid repair with antegrade stent delivery to the descending thoracic aorta. J. Thorac. Cardiovasc. Surg..

[72] Canaud L., Gandet T., Sfeir J., Ozdemir B.A., Solovei L., Alric P. (2019). Risk factors for distal stent graft-induced new entry tear after endovascular repair of thoracic aortic dissection. J. Vasc. Surg..

[73] Melloni A., Kahlberg A., Rinaldi E., Bilman V., Favia N., Melissano G., Chiesa R. (2022). Open Thoracoabdominal Aortic Procedures following Endovascular Intervention. AORTA.

[74] Fehrenbacher J.W., Corvera J.S. (2012). Best surgical option for thoracoabdominal aneurysm repair-the open approach. Ann. Cardiothorac. Surg..

[75] Bernal L.R., Afifi R.O., Estrera A.L. (2024). Open repair with latissimus muscle flap coverage for treatment of infected thoracic endovascular aneurysm repair. J. Vasc. Surg. Cases Innov. Tech..

[76] Tsuge I., Saito S., Yamazaki K., Sakamoto K., Tsunoda S., Katsube M., Arata J., Sakamoto M., Minatoya K., Morimoto N. (2020). Latissimus Dorsi Muscle Flap with a Distally Based Serratus Anterior Extension for Salvaging Aortic Graft Infection. Plast. Reconstr. Surg. Glob. Open..

[77] Okita Y., Yamanaka K., Okada K., Matsumori M., Inoue T., Fukase K., Sakamoto T., Miyahara S., Shirasaka T., Izawa N. (2014). Strategies for the treatment of aorto-oesophageal fistula. Eur. J. Cardiothorac. Surg..

[78] Afifi R.O., Mushtaq H.H., Sandhu H.K., Khalil K., Safi H.J., Estrera A.L. (2016). Successful Multistaged Surgical Management of Secondary Aortoesophageal Fistula with Graft Infection. Ann. Thorac. Surg..

[79] Obeid T., Hicks C.W., Yin K., Arhuidese I., Nejim B., Kilic A., Black J.H., Malas M. (2016). Contemporary outcomes of open thoracoabdominal aneurysm repair: Functional status is the strongest predictor of perioperative mortality. J. Surg. Res..

